# Diagnostic and prognostic value of blood inflammation and biochemical indicators for intrahepatic cholestasis of pregnancy in Chinese pregnant women

**DOI:** 10.1038/s41598-022-22199-9

**Published:** 2022-12-02

**Authors:** Mengjun Luo, Li Wang, Haibo Yao, Yizhou Wen, Dengcheng Cao, Wei Shen, Chenggui Liu

**Affiliations:** 1 Department of Clinical Laboratory, School of Medicine, Chengdu Women’s and Children’s Central Hospital, University of Electronic Science and Technology of China, No.1617 Ri Yue Street, Chengdu, 611731 Sichuan China; 2 Department of Medical Records, School of Medicine, Chengdu Women’s and Children’s Central Hospital, University of Electronic Science and Technology of China, Chengdu, 611731 Sichuan China; 3 Department of Pediatric Cardiology, School of Medicine, Chengdu Women’s and Children’s Central Hospital, University of Electronic Science and Technology of China, Chengdu, 611731 Sichuan China

**Keywords:** Endocrine system and metabolic diseases, Metabolic disorders

## Abstract

Intrahepatic cholestasis of pregnancy (ICP) is a common liver disease during pregnancy, that has serious complications. This study aimed to compare the blood inflammation and biochemical markers of pregnant women with ICP in Southwest China and analyse their diagnostic value for ICP. A controlled cross-sectional study was conducted, and routine blood and biochemical indicators of 304 diagnosed ICP patients and 363 healthy pregnant women undergoing routine prenatal examination were assessed. The blood inflammatory indicators and biochemical indicators were compared between the ICP groups and normal groups. In this study, the levels of the ALT, AST, GGT, TBIL and DBIL biochemical indicators and the levels of WBC, neutrophils, NLR and PLR inflammatory indicators in the ICP group were significantly higher than those in healthy pregnant women (*p* < 0.001). The PA and lymphocytes of the ICP group were significantly lower than those of the normal group (*p* < 0.001). ROC curves showed that ALT and the NLR had higher predictive value for ICP. The GGT, TBA and NLR of pregnant women with ICP in the preterm group were significantly higher than those in the term group, and the combined NLR and TBA had a certain predictive value for preterm birth.

## Introduction

Intrahepatic cholestasis of pregnancy (ICP) is an idiopathic disease of pregnancy that occurs in the middle and third trimesters of pregnancy. It is characterized by mild to severe skin pruritus and abnormal liver function. Postpartum, symptoms are rapidly relieved, and liver function returns to normal^1^. The incidence of ICP worldwide is between 0.2 and 25%. In China, the incidence of ICP is between 2.3 and 6.0%, and the recurrence rate in a second pregnancy is 40% and 60%^2,3^. At present, the exact cause of ICP is not clear, but heredity, hormone, nutrition and environmental factors may be related to it^4^. In clinical practice, the underdiagnosis, misdiagnosis and untimely treatment of ICP without obvious causes still occur from time to time. Long-term high bile acid levels will cause obvious vasospasm on the surface of placental villi, reduce blood flow through the intervillous area of the placenta, and eventually lead to fetal insufficiency and complications of the mother and fetus^5–7^. The pathogenesis of ICP is often accompanied by liver injury and changes in liver enzyme levels to varying degrees, and abnormal levels of γ-glutamyl transferase (GGT) and bile acid can support the diagnosis of ICP^8^. Therefore, the early diagnosis, screening and treatment of ICP is very important. For pregnant women with no obvious cause of skin pruritus during pregnancy, it is very important to use new detection indicators for diagnosis.

The exact pathogenesis of ICP is complex, and inflammation may play an important role^9^. The increase in the pathological concentration of total bile acid (TBA) in the pathogenesis of ICP can induce hepatocyte cells to produce inflammatory response and induce a variety of inflammatory response to promote the infiltration of neutrophils, lymphocytes and macrophages in the blood^9,10^. It has been reported that the levels of inflammatory factors, such as Iinterleukin-6 (IL-6), interleukin-17 (IL-17) and tumor necrosis factor α (TNF-α) are elevated in parturient women with ICP are significantly higher than those in normal pregnant women. These elevated inflammatory markers disrupt the immune balance between the mother and fetus, thus reflecting the severity of the condition^11–13^. The white blood cell (WBC), neutrophil-to-lymphocyte ratio (NLR), and platelet-to-lymphocyte ratio (PLR) can be used as indicators of inflammation and are widely used in the prediction of gestational diabetes mellitus, atherosclerosis and tumors^14,15^. However, the diagnostic value of the above indicators for ICP is unknown. In this study, we explored the predictive value of routine blood and biochemical indicators for ICP.

## Results

### Study population

The baseline characteristics of the 667 included pregnant women are shown in Table1. Notably, the serum levels of ALT (alanine aminotransferase), AST (aspartate aminotransferase), GGT (γ-glutamyl transferase), TBIL (total bilirubin), DBIL (direct bilirubin) and TBA (total bile acid) and the WBC (white blood cell) count, neutrophil count, NLR (neutrophil-to-lymphocyte ratio) and PLR (platelet-to-lymphocyte ratio) in pregnant women with ICP were significantly higher than those in healthy pregnant women. However, the levels of PA (prealbumin) and lymphocytes in pregnant women with ICP were significantly lower than those in healthy pregnant women (*p* < *0.001*, see Table 1).

**Table 1:** Comparison of general information between the ICP and healthy pregnant groups.

Variables	Normal (N = 363)	ICP (N = 304)	*p-value*
Age (years)	30.04 ± 4.00	29.60 ± 4.28	0.126
Gestational week	34.08 ± 2.89	34.03 ± 3.89	0.091
ALT (U/L)	16.80 (13.00, 24.30)	33.60 (30.20, 38.85)	< 0.001
AST (U/L)	19.00 (16.10, 22.90)	43.20 (19.93, 85.00)	< 0.001
GGT (U/L)	13.60 (9.30, 21.20)	21.50 (9.62, 39.52)	< 0.001
PA (g/L)	206.00 (191.10, 221.50)	183.80 (148.02, 212.85)	< 0.001
TBIL (μmol/L)	5.70 (4.40,7.50)	7.50 (5.40, 10.17)	< 0.001
DBIL (μmol/L)	2.00 (1.60, 2.40)	3.00 (2.02, 4.60)	< 0.001
TBA (mmol/L)	2.10 (1.80, 4.40)	19.50 (13.75, 31.70)	< 0.001
WBC × 10^9^/L	7.28 (6.22, 8.63)	7.98 (6.68, 9.18)	< 0.001
PLT × 10^9^/L	166.00 (133.00, 206.00)	172.00 (138.00, 215.00)	0.062
Neutrophils × 10^9^/L	5.22 (4.33, 6.32)	5.81 (4.83, 7.04)	< 0.001
Lymphocytes × 10^9^/L	1.62 (1.42, 1.86)	1.37 (1.16, 1.59)	< 0.001
NLR	3.23 (2.74, 3.83)	4.08 (3.39, 5.39)	< 0.001
PLR	101.09 (80.19, 123.91)	126.36 (96.26, 162.48)	< 0.001

ALT, alanine aminotransferase; AST, aspartate aminotransferase; GGT, γ-glutamyl transferase; PA, prealbumin; TBIL, total bilirubin; DBIL, direct bilirubin; TBA, bile acid; WBC, white blood cell count; PLT, platelets; NLR, neutrophil-to-lymphocyte ratio; PLR, platelet-to-lymphocyte ratio.

We further compared the biochemical and blood inflammatory indicators among the mild ICP group, the severe ICP group and the normal group. We found that except for platelets, the biochemical and blood inflammatory indicators in the mild and severe groups were all significantly different from those in the normal group (*p* < *0.001*, shown in Table 2). The TBA level in the severe ICP group was higher than that in the mild ICP group. However, there were no significant differences in other biochemical or inflammatory indicators between the two groups (shown in Table 2).

**Table 2:** Clinical characteristics of the pregnant women with mild and severe ICP and normal pregnant women.

Variables	Normal (N = 363)	Mild ICP (N = 262)	Severe ICP (N = 42)	*p-value*
Age (years)	30.04 ± 4.00	29.48 ± 4.29	30.31 ± 4.18	0.095
Gestational week	34.08 ± 2.89	34.18 ± 3.75*	33.10 ± 4.62^#^	0.030
ALT (U/L)	16.80 (13.00, 24.30)	33.60 (30.37, 38.35)*	33.85 (29.75, 58.85)*	< 0.001
AST (U/L)	19.00 (16.10, 22.90)	43.20 (20.22, 82.12)*	44.05 (17.00, 104.20)*	< 0.001
GGT (U/L)	13.60 (9.30, 21.20)	22.00(9.92, 38.55)*	16.15 (8.90, 49.92)*	< 0.001
PA (g/L)	206.00 (191.10, 221.50)	184.35(146.20, 212.90)*	179.05 (149.40, 211.52)*	< 0.001
TBIL (μmol/L)	5.70 (4.40, 7.50)	7.40 (5.40, 10.10)*	7.80 (5.55, 10.35)*	< 0.001
DBIL (μmol/L)	2.00 (1.60, 2.40)	3.00 (2.00, 4.50)*	3.50 (2.30, 6.47)*	< 0.001
TBA (mmol/L)	2.10 (1.80, 4.40)	17.75 (13.27, 24.90)*	52.00 (44.75, 80.95)*^#^	< 0.001
WBC × 10^9^/L	7.28 (6.22, 8.63)	8.02(6.67, 9.28)*	7.44 (6.64, 8.37)	< 0.001
PLT × 10^9^/L	166.00 (133.00, 206.00)	171.00 (137.75, 215.50)	178.00 (139.25, 211.75)	0.154
Neutrophils × 10^9^/L	5.22 (4.33, 6.32)	5.87 (4.87, 7.08)*	5.26 (4.55, 6.23)	< 0.001
Lymphocytes × 10^9^/L	1.62 (1.42, 1.86)	1.36 (1.15, 1.59)*	1.41 (1.18, 1.62)*	< 0.001
NLR	3.23 (2.74, 3.83)	4.08 (3.47, 5.45)*	4.05 (2.90, 5.22)*	< 0.001
PLR	101.09 (80.19, 123.91)	125.75 (96.99, 161.50)*	128.49 (98.23, 170.28)*	< 0.001

ALT, alanine aminotransferase; AST, aspartate aminotransferase; GGT, γ-glutamyl transferase; PA, prealbumin; TBIL, total bilirubin; DBIL, direct bilirubin; TBA, bile acid; WBC, white blood cell count; PLT, platelets; NLR, neutrophil-to-lymphocyte ratio; PLR, platelet-to-lymphocyte ratio.

*, *p* < *0.05*, mild ICP/severe ICP compared with normal pregnancy;^#^, *p* < *0.05*, severe ICP compared with mild ICP.

### Correlation analysis between TBA and blood inflammatory indicators

We found that the TBA level in pregnant women with ICP was positively correlated with the WBC (r = 0.113, *p* = *0.003*), neutrophil count (r = 0.130, *p* = *0.001*), NLR (r = 0.267, *p* < *0.001*) and PLR (r = 0.248, *p* < *0.001*) and negatively correlated with the lymphocyte count (r = -0.204, *p* < 0.001).

### Predictive value of biochemical and blood inflammation indicators for ICP

**Figure 1:**
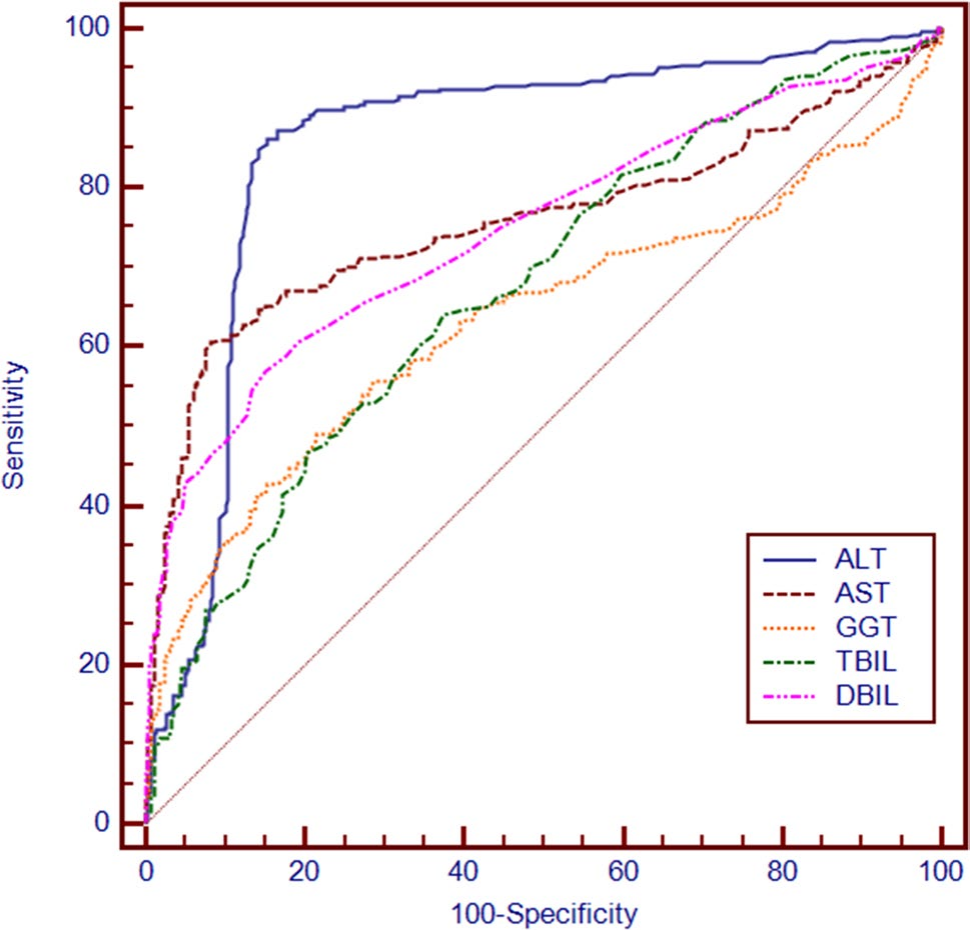
ROC curves of the predictors of biochemical indicators for ICP. ALT, alanine aminotransferase; AST, aspartate aminotransferase; GGT, γ-glutamyl transferase; TBIL, total bilirubin; DBIL, direct bilirubin.

We used ROC curves to analyse the predictive value of the biochemical indicators and found that ALT, AST, GGT, TBIL and DBIL levels were significantly different between the ICP and normal groups. Among the biochemical indicators, ALT (AUC = 0.855, 95% confidence interval (CI): 0.825, 0.880) had the highest predictive value for ICP (p < 0.001, see Table 3, Fig. 1). The optimal cut-off value of ALT for diagnosing ICP was 28.0 U/L, with high sensitivity (86.14%) and specificity (84.57%) (see Table 3, Fig. 1).

**Table 3:** Prediction performance of the parameters for biochemistry indicators.

Variables	AUC	95%*CI*	Sensitivity (%)	Specificity (%)	Cutoff value	*p-value*
ALT (U/L)	0.855	0.825–0.880	86.14	84.57	28.0	< 0.001
AST (U/L)	0.757	0.722–0.789	60.40	91.74	30.2	< 0.001
GGT (U/L)	0.633	0.595–0.670	49.01	78.51	22.3	< 0.001
TBIL (μmol/L)	0.673	0.636–0.709	64.14	62.53	6.4	< 0.001
DBIL (μmol/L)	0.747	0.712–0.780	56.91	84.85	2.7	< 0.001

ALT, alanine aminotransferase; AST, aspartate aminotransferase; GGT, γ-glutamyl transferase; TBIL, total bilirubin; DBIL, direct bilirubin.

**Figure 2:**
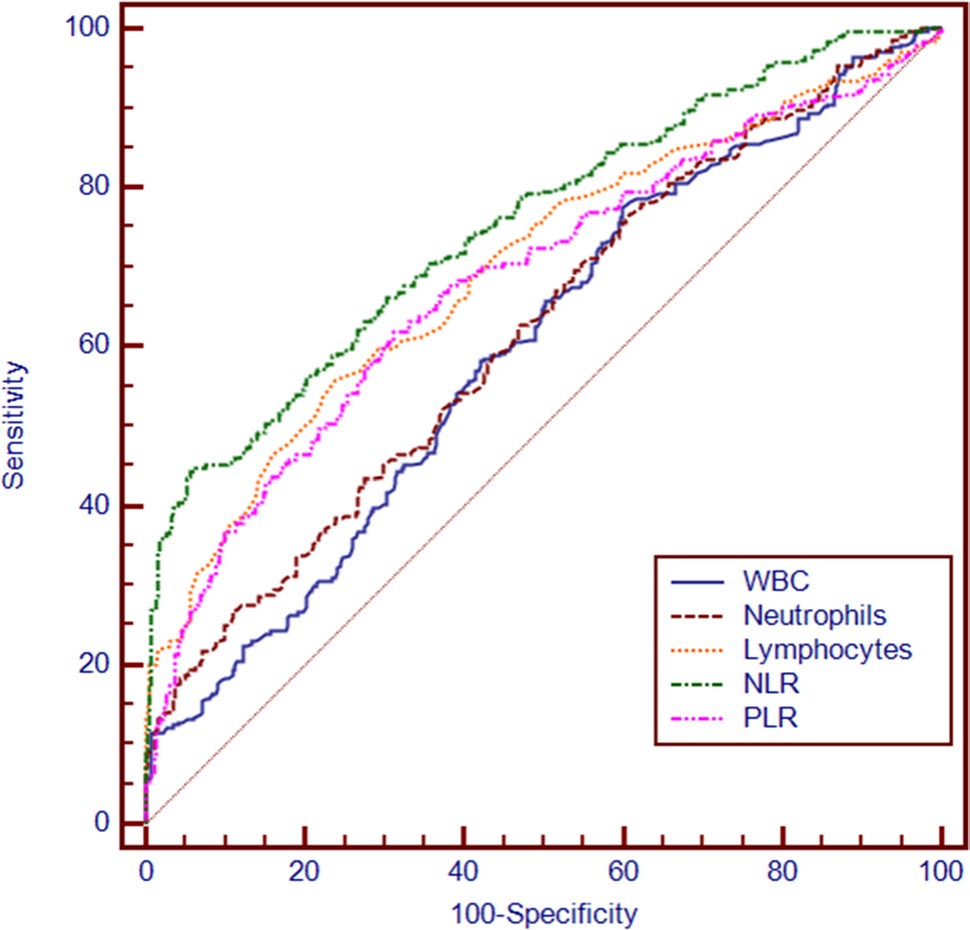
ROC curves of the predictors of blood inflammatory indicators for ICP. WBC, white blood cell count; NLR, neutrophil-to-lymphocyte ratio; PLR, platelet-to-lymphocyte ratio.

Similarly, we analyzed the predictive value of blood inflammatory indicators for ICP using ROC curves, and found that the WBC count, neutrophil count, lymphocyte count, NLR and PLR were significantly different between the ICP group and normal groups (see Table4). Predictive analysis of ROC curves based on inflammatory indicators showed that the NLR (AUC = 0.753) had certain predictive value for ICP with a specificity of 94.21% and sensitivity of 44.41%. This predictive value was higher than that of the WBC count, neutrophil count, lymphocyte count and PLR (see Table 4, Fig. 2).

**Table 4:** Prediction performance of the parameters for blood inflammatory indicators.

Variables	AUC	*95%CI*	Sensitivity (%)	Specificity (%)	Cutoff value	*p-value*
WBC	0.600	0.561–0.637	77.63	39.94	6.54	< 0.001
neutrophils	0.620	0.581–0.657	26.97	88.98	6.92	< 0.001
lymphocytes	0.699	0.662–0.733	55.92	76.03	1.41	< 0.001
NLR	0.753	0.718–0.785	44.41	94.21	4.38	< 0.001
PLR	0.682	0.645–0.718	61.84	68.87	115.66	< 0.001

WBC, white blood cell count; NLR, neutrophil-to-lymphocyte ratio; PLR, platelet-to-lymphocyte ratio.

### Multivariate analysis of independent factors predicting ICP with inflammatory indicators

Following adjustment for age and gestational weeks, the multivariate logistic regression model showed that the WBC count, neutrophil count, lymphocyte count, NLR and PLR were independent risk factors for ICP (*p* < 0.05, shown in Table 5).

**Table 5:** Independent risk factors predicting ICP by blood inflammatory factors.

Variables	OR	*95%CI*	*p-value*
WBC	43.676	15.672–121.720	< 0.001
neutrophils	0.015	0.004–0.049	< 0.001
lymphocytes	0.029	0.003–0.287	0.003
NLR	3.282	1.425–7.556	0.005
PLR	1.007	1.001–1.012	0.012

WBC, white blood cell count; NLR, neutrophil-to-lymphocyte ratio; PLR, platelet-to-lymphocyte ratio.

### Predictive value of biochemical and blood inflammation indicators for preterm delivery in the ICP group

**Figure 3:**
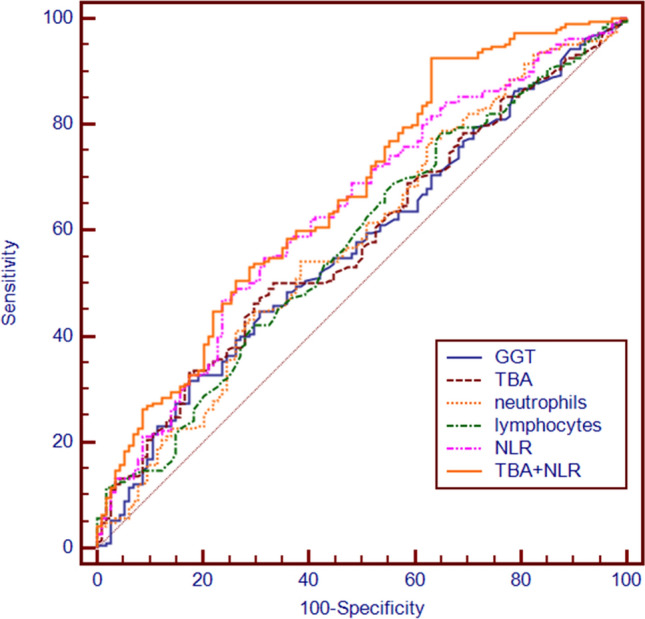
ROC curves of the predictors of the NLR and GGT level for preterm delivery. GGT, γ-glutamyl transferase; TBA, bile acid; NLR, neutrophil-to-lymphocyte ratio; PLR, platelet-to-lymphocyte ratio.

There were significant differences in the levels of GGT, TBA, neutrophils, lymphocytes and NLR between the ICP preterm subgroup (GW < 37 weeks) and the ICP full-term subgroup (GW ≥ 37 weeks) (*p* < 0.05, shown in Table 6). The results of ROC analysis of the above differential inflammatory indicators and biochemical indicators showed that the NLR blood inflammatory indicator and the TBA biochemical indicator had some predictive value for preterm delivery. The area under the curve of the NLR was 0.635 (95% confidence interval (CI): 0.571, 0.700), and the area under the curve of TBA levels was 0.579 (95% confidence interval (CI): 0.514, 0.664). Multivariate logistic regression analysis was used to assess the diagnostic efficacy of the NLR and TBA levels for the combined diagnosis of ICP. The combined diagnosis of NLR and TBA levels increased the area under the curve (AUC = 0.669) and the sensitivity (92.63%) of diagnosis (shown in Table 7, Fig. 3).

**Table 6:** Clinical characteristics of the preterm delivery and full-term delivery groups of pregnant women with ICP.

Variables	Preterm delivery (n = 190)	Full-term delivery (n = 114)	*p-value*
Age (years)	29.71 ± 4.39	29.41 ± 4.09	0.204
Gestational week	34.05 ± 1.22	37.55 ± 0.692	< 0.001
ALT (U/L)	33.70 (30.20, 39.00)	33.55(30.28, 38.95)	0.582
AST (U/L)	51.10 (20.88, 96.30)	35.50(18.08, 71.13)	0.057
GGT (U/L)	23.35 (11.00, 47.00)	19.10 (8.88, 34.43)	0.041
PA (U/L)	186.55 (148.80, 216.03)	177.80 (145.10, 207.50)	0.267
TBIL (μmol/L)	7.65 (5.40, 10.33)	7.20 (5.48, 9.33)	0.625
DBIL (μmol/L)	3.05 (2.10, 5.10)	2.90 (2.00, 4.23)	0.340
TBA (mmol/L)	20.70 (14.38, 33.48)	18.40(13.28, 26.05)	0.021
WBC × 10^9^/L	8.05 (6.87, 9.19)	7.89 (6.41, 9.19)	0.187
PLT × 10^9^/L	174.50 (136.00, 217.75)	169.00 (143.00, 213.50)	0.933
Neutrophils × 10^9^/L	6.09 (4.96, 7.09)	5.62 (4.58, 6.70)	0.019
Lymphocytes × 10^9^/L	1.35 (1.12, 1.56)	1.42 (1.19, 1.68)	0.025
NLR	4.42 (3.62, 5.69)	3.79 (3.12, 5.53)	< 0.001
PLR	131.19 (99.85, 164.16)	123.68 (91.55, 153.06)	0.098

ALT, alanine aminotransferase; AST, aspartate aminotransferase; GGT, γ-glutamyl transferase; PA, prealbumin; TBIL, total bilirubin; DBIL, direct bilirubin; TBA, bile acid; WBC, white blood cell count; PLT, platelets; NLR, neutrophil-to-lymphocytes ratio; PLR, platelet-to-lymphocyte ratio.

**Table 7:** Prediction performance of the parameters on preterm delivery in pregnant women with ICP.

Variables	AUC	*95%CI*	Sensitivity (%)	Specificity (%)	Cutoff value	*p-value*
GGT	0.570	0.477–0.613	44.70	69.30	28.20	0.041
TBA	0.579	0.514–0.644	47.40	69.30	21.70	0.021
neutrophils	0.580	0.514–0.647	54.21	61.40	5.87	0.019
lymphocytes	0.577	0.511–0.543	78.40	35.10	1.57	0.025
NLR	0.635	0.571–0.700	54.70	68.40	4.22	< 0.001
NLR + TBA	0.669	0.605–0.732	92.63	36.84	0.501	< 0.001

GGT, γ-glutamyl transferase; TBA, total bile acid; NLR, neutrophil-to-lymphocyte ratio.

## Discussion

ICP is a hepatic disease associated with pregnancy and is characterized by pruritus and concomitant liver dysfunction and typically increased serum levels of total bile acid^16^. In recent years, the implementation of the two-child policy in China has led to an increase in the proportion of pregnant women with advanced maternal age, with a concomitant increase in the incidence of ICP. The incidence of ICP has significant geographical characteristics, with a prevalence of 1.0–4.0% in the Yangtze River region and a high incidence in Sichuan, China^17,18^. The etiology of ICP is multifactorial, estrogen-bile acid axis and immunological factors like inflammatory factors may underlie the effect of cholestasis effect on hepatocytes^19^. Because of the great influence of ICP on the fetus, it can lead to the occurrence of adverse pregnancy outcomes, such as preterm delivery and fetal distress^1^. Therefore, research on its mechanism has become increasingly popular, and early screening and diagnosis of the disease during pregnancy is increasingly important. Most of the current studies support that inflammatory mediators are involved in the development of ICP and that stagnant bile acid directly activates the inflammatory signaling pathway in the liver, promoting the development of inflammatory reactions and the accumulation of proinflammatory mediators that further lead to liver injury^10,20,21^. Therefore, ICP is often accompanied by varying degrees of abnormalities in inflammatory factors and liver enzyme levels^22^.

At present, the laboratory diagnosis of ICP is mainly performed by using serum TBA. However, the diagnostic cut-off values of TBA also vary greatly according to the measurement method used, fasting status, study population and gestational age at the time of diagnosis, so screening for appropriate clinical indicators can be crucial as an adjunct to ICP diagnosis^22–24^. Previous studies have demonstrated that bile acids have some cytotoxic effects, causing cell apoptosis and necrosis and leading to increased levels of ALT and TBIL. Additionally, bilirubin can also interact with bile and aggravate hepatocyte damage, leading to abnormal elevation of biochemical indicators^25^. In clinical practice, changes in biochemical indicators such as liver enzymes are important markers of hepatocyte injury and are also widely used^26,27^. To determine the diagnostic value of biochemical indicators for ICP, we evaluated the diagnostic efficacy of liver function indicators for ICP. In the ICP group, the levels of ALT, AST, GGT, TBIL and DBIL were significantly higher than those in the healthy group, and ROC curve analysis showed that ALT had higher a predictive value for ICP with a sensitivity of 86.14% and a specificity of 84.57%. This finding is consistent with previous studies^28^. The above results confirm that blood biochemical indicators have a certain predictive value for ICP. For pregnant women who lack a high level of TBA, the combination of biochemical indicators and clinical symptoms may be helpful for the diagnosis of ICP.

It has been reported that the levels of inflammatory factors can be used to assess the degree of abnormal liver function in patients with ICP. It may participate in the process of liver injury and disrupt the immune balance between the mother and fetus, thus indicating the severity of the condition^19,21,29^. However, the specific role of inflammation in the pathogenesis of ICP is not known. Previous studies have reported that cytokines IL-8, IL-10 and IL-12 play a certain role in the pathogenesis of ICP, but the above indicators cannot be widely used in clinical diagnosis in developing countries due to technical and application problems^21,30,31^. To this end, we evaluated the diagnostic value of routine blood examination in routine clinical obstetrical examination for ICP. In our study, we found that the numbers of WBC and neutrophils, the NLR and the PLR were significantly higher in the ICP group than in the normal group, while the lymphocyte levels was significantly lower in the ICP group than in the normal group (shown in Table 1). By correlation analysis, we found that TBA was positively correlated with the WBC count, neutrophil count, NLR and PLR and negatively correlated with lymphocyte count. Furthermore, our study also confirmed that the WBC count, neutrophil count, lymphocyte count, NLR and PLR are independent risk factors for ICP, and ROC curve analysis confirmed that the NLR had a higher diagnostic value for ICP (shown in Tables 4 and 5). The above changes are consistent with the reports in the literature^21^. The above results suggest that the elevation of TBA is consistent with the elevation of routine blood inflammatory indicators and that the occurrence of ICP may be directly related to inflammation.

Although ICP has little risk for mothers, it is related to adverse perinatal outcomes, leading to preterm birth, stillbirth^32^. Preterm birth is a common clinical complication of ICP. Cohort studies have confirmed that an increase in the maternal serum TBA concentration will increase the incidence of spontaneous preterm birth^33^. Long-term low-grade chronic inflammation increases the risk of preterm birth^34^. Therefore, we analyzed the blood inflammation and biochemical indicators of preterm and term births in pregnant women with ICP. We found that GGT, TBA, neutrophils and NLR of preterm delivery in pregnant women with ICP were higher than full-term delivery groups. We used ROC curves to predict the diagnostic value of the abovementioned indicators for preterm birth in pregnant women with ICP. The NLR had certain predictive value for preterm birth, but the sensitivity and specificity were not high enough. However, the combined detection of the NLR and TBA level increases the predictive efficacy and sensitivity for preterm birth in ICP. The mechanisms of preterm delivery in ICP are still unclear. The accumulation of the inflammatory response induced by ICP, which leads to the progression of ICP, and the accumulation of TBA may be involved in the onset of preterm delivery^35,36^.

ICP is the most common pregnancy-specific liver disease^37^. The current clinical grading criteria for ICP are mainly based on the level of serum TBA^38^. In this study, we considered that the critical value of TBA for diagnosing ICP was 10 µmol/L; the TBA level of 10–40 µmol/L was defined as mild ICP, and the TBA level > 40 µmol/L was defined as severe ICP, as suggested in the literature^30^. As serum TBA could not be used to distinguish the ICP patients with low pruritus from normal pregnant women, and there is no consensus on the diagnostic threshold of liver enzymes, which may limit the early diagnosis of ICP^21,30,32^. Since the pathogenesis of ICP is closely related to inflammation, in our study, we used routine blood tests, which are commonly used in clinical practice, for the ancillary diagnosis of ICP and its complications leading to preterm delivery. Our study confirmed that biochemical indicators and blood inflammatory indicators are related to ICP and its complications in preterm delivery. Some indicators can be used alone or in combination for the prediction and auxiliary diagnosis of ICP. For patients with clinical ICP symptoms and a lack of typical symptoms, multi-indicator combined detection should be used to reduce the occurrence of complications.

## Materials and methods

### Study subjects

This study was a cross-sectional analysis of 304 pregnant women with diagnosed ICP and 363 normal pregnant women in Chengdu Women’s and Children’s Central Hospital, School of Medicine, University of Electronic Science and Technology of China, from January 2020 to June 2021. The inclusion criteria of the ICP group were as follows^39^: (1) pregnant women with pruritus without rash and elevated TBA level ≥ 10 µmol/L and/or aminotransferase levels detectable in their blood sample; (2) patients were classified as having mild or severe cholestasis based on TBA concentrations of 10–40 or ≥ 40 µmol/L, respectively (3) patients without diabetes, hepatobiliary diseases, immunological diseases or other pregnancy complications; (4) patients whose gestational age was determined based on the first day of the last menstrual period and/or first trimester ultrasonographic measurements; (5) patients with a singleton pregnancy confirmed by ultrasound; (6) patients with voluntary participation; and (7) patients with preterm delivery, defined as delivery before 37 completed weeks of gestation.

### Data collection

All venous blood samples were collected from peripheral vein blood in a vacutainer tube after the participants had fasted for 12 h. The hemogram samples were collected in ethylenediaminetetraacetic acid (EDTA) anticoagulant tubes, and blood serum samples were collected into standard gel separator tubes for biochemical tests. All samples were detected within 1 h after collection. Alanine aminotransferase (ALT), aspartate aminotransferase (AST), γ-glutamyl transferase (GGT), total bilirubin (TBIL), direct bilirubin (DBIL), prealbumin (PA) and total bile acid (TBA) levels were measured by standard laboratory techniques using a Hitachi automatic analyzer (Hitachi 7600 automatic analyzer, Tokyo, Japan). The CBC included the white blood cell (WBC) count, platelet (PLT) count, neutrophil count, and lymphocyte count. The NLR was calculated as the neutrophil count divided by the lymphocyte count, and the PLR was calculated as the platelet count divided by the lymphocyte count. All CBC analyses were performed with a Sysmex Hemato analyzer (Sysmex XN-1000 Hemato analyzer, Kobe, Japan).

### Statistical analysis

All data were analyzed by SPSS 22.0 (IBM, Chicago, USA). The normally distributed measurement data are expressed as the mean ± standard deviation, and the measurement data that did not conform to a normal distribution are expressed as the median (interquartile range (IQR)). The Mann‒Whitney U test was used to perform comparisons between groups. Receiver operating characteristic (ROC) analysis was used to identify the best cut-off value, and the sensitivity and specificity of each indicator for ICP diagnosis were screened. Spearman correlation coefficients were computed to examine the association between TBA and blood inflammatory indicator levels. A multivariate logistic regression model was used to analyse the risk factors for ICP and the diagnostic value of the combined indices. A *p* < 0.05 was considered statistically significant.

### Ethical approval

This study was approved by the ethics committee of the Women and Children Affiliated Hospital of the Medical College of the University of Electronic Science and Technology, and clinically informed written consent was obtained from each patient. All methods were carried out in accordance with the Declaration of Helsinki.

### Limitations

Our study provides evidence that blood inflammatory indicators and biochemical indicators have a direct correlation with ICP. At present, the method of diagnosing ICP based on serum inflammatory markers and biochemical markers still needs to be studied, and larger and further prospective studies to evaluate the use of the markers and in-depth mechanistic studies of ICP are needed.

## Data Availability

The authors confirm that all the data-based findings are fully available without restriction. All relevant data are included in the paper and references. The datasets used and/or analysed during the current study are available from the corresponding author upon reasonable request.
