# Publisher Correction: Diagnostic and prognostic value of blood inflammation and biochemical indicators for intrahepatic cholestasis of pregnancy in Chinese pregnant women

**DOI:** 10.1038/s41598-022-26533-z

**Published:** 2022-12-19

**Authors:** Mengjun Luo, Li Wang, Haibo Yao, Yizhou Wen, Dengcheng Cao, Wei Shen, Chenggui Liu

**Affiliations:** 1 Department of Clinical Laboratory, School of Medicine, Chengdu Women’s and Children’s Central Hospital, University of Electronic Science and Technology of China, No.1617 Ri Yue Street, Chengdu, 611731 Sichuan China; 2 Department of Medical Records, School of Medicine, Chengdu Women’s and Children’s Central Hospital, University of Electronic Science and Technology of China, Chengdu, 611731 Sichuan China; 3 Department of Pediatric Cardiology, School of Medicine, Chengdu Women’s and Children’s Central Hospital, University of Electronic Science and Technology of China, Chengdu, 611731 Sichuan China

Correction to: *Scientific Reports* 10.1038/s41598-022-22199-9, published online 02 December 2022

In the original version of this Article, Mangjun Luo was incorrectly marked as a corresponding author. The correct corresponding authors for this Article are Li Wang and Chenggui Liu.

In addition, Mengjun Luo and Li Wang were omitted as equally contributing authors.

The original Article has been corrected.

